# Effect of neoadjuvant chemotherapy in patients with gastric cancer: a PRISMA-compliant systematic review and meta-analysis

**DOI:** 10.1186/s12885-018-4027-0

**Published:** 2018-01-31

**Authors:** Zhi-Feng Miao, Xing-Yu Liu, Zhen-Ning Wang, Ting-Ting Zhao, Ying-Ying Xu, Yong-Xi Song, Jin-Yu Huang, Hao Xu, Hui-Mian Xu

**Affiliations:** 1 Department of Surgical Oncology, First Hospital of China Medical University, Shenyang, 110001 China; 2 Department of Breast Surgery, First Hospital of China Medical University, Shenyang, China

**Keywords:** Gastric cancer, Neoadjuvant chemotherapy, Meta-analysis, Overall aurvival, Prognosis

## Abstract

**Background:**

Neoadjuvant chemotherapy (NAC) is extensively used in the treatment of patients with gastric cancer (GC), particularly in high risk, advanced gastric cancer. Previous trials testing the efficacy of NAC have reported inconsistent results.

**Methods:**

This study compares the combined use of NAC and surgery with surgery alone for GC by using a meta-analytic approach. We performed an electronic search of PubMed, EmBase, and the Cochrane Library to identify randomized controlled trials (RCTs) on NAC published before Oct 2015. The primary outcome of the studies was data on survival rates for patients with GC. The summary results were pooled using the random-effects model. We included 12 prospective RCTs reporting data on 1538 GC patients.

**Results:**

Patients who received NAC were associated with significant improvement of OS (*P* = 0.001) and PFS (*P* < 0.001). Furthermore, NAC therapy significantly increased the incidence of 1-year survival rate (SR) (*P* = 0.020), 3-year SR (*P* = 0.011), and 4-year SR (*P* = 0.001). Similarly, NAC therapy was associated with a lower incidence of 1-year (*P* < 0.001), 2-year (*P* < 0.001), 3-year (*P* < 0.001), 4-year (*P* = 0.001), and 5-year recurrence rate (*P* = 0.002). Conversely, patients who received NAC also experienced a significantly increased risk of lymphocytopenia (*P* = 0.003), and hemoglobinopathy (*P* = 0.021).

**Conclusions:**

The findings of this study suggested that NAC is associated with significant improvement in the outcomes of survival and disease progression for GC patients while also increasing some toxicity.

## Background

Although cancer-related incidence and mortality have been decreasing in the past few years, gastric cancer (GC) remains the fourth most common malignancy in world [1]. The incidence of early gastric cancer were highest in China, Japan, and Korea, which accounting for greater than 50% of the world totals [2]. The prognosis of GC patients is determined relative to their cancer stage. Such as, for patients with advanced stages of GC (III and IV preoperative TNM staging), the 5-year survival rate of approximately 25 % [3]. It is estimated that local recurrence or distant metastases will happen in about 60% of GC patients even if they undergo macroscopic resection [4].

Multimodality therapy including neoadjuvent chemotherapy (NAC) therapy and D1+/D2 gastrectomy is regarded as standard of care across Europe and Australasia and is increasing accepted in North America [5]. D2 Gastrectomy with adjuvant therapy is practised routinely in Asia, whereas patients with advanced gastric tumors needed to received NAC therapy [3]. A previous meta-analysis of 6 randomized controlled trial (RCT) has no significant effect on overall survival or complete resection [6]. However, one trial [7] was included in a previous meta-analysis despite its use of imbalanced postoperative chemotherapy, resulting in obvious bias [6]. Additionally, a misjudged trial [8] for RCT and two researches [9, 10] with unmatched postoperative treatment led to an ineligible criteria in Ge’s analysis [11]. Moreover, the meta-analysis of Li et al. [12] and Wu et al. [13] included non-RCTs and few qualified RCTs. Finally, the potential role of NAC as treatment in patients with GC on year-specific survival rate has not been investigated by previous meta-analyses.

In order to reach a higher level of meta-analysis, the pooled data for this study will consist entirely of RCTs. Using only these qualified RCTs, we carried out a meta-analysis and systemic review to demonstrate the survival outcomes related to NAC.

## Methods

### Data sources, search strategy, and selection criteria

This review was conducted and reported according to the Preferred Reporting Items for Systematic Reviews and Meta-Analysis Statement issued in 2009 [14]. Ethics approval was not necessary for this study, as only de-identified pooled data from individual studies were analyzed. Following the Cochrane Handbook for systematic review and meta-analysis, electronic databases including the Cochrane online library, PubMed and Embase were utilized for the comprehensive search, and the following terms were used for the identification of relevant trials: (“gastric cancer” OR “gastric carcinoma” OR “gastric neoplasm” OR “stomach cancer” OR “stomach neoplasm” OR “stomach carcinoma” OR “gastroesophageal junction neoplasm” OR “cancer of stomach”) AND (“neoadjuvant chemotherapy” OR “preoperative chemotherapy”). We also conducted manual searches of reference lists from all relevant original research and review articles to identify additional eligible studies. The medical subject heading, methods, patient population, design, intervention, control, and outcome variables of these articles were used to identify relevant studies.

We introduced a two-stage process to select eligible studies based on the above eligibility criteria. Studies selected via systematic identification were evaluated for consistency through their title, abstract and full text, and those that failed to meet the inclusion criteria were rejected. For the articles with only the abstract available, we tried to contact the corresponding author in an effort to obtain the full text. Trials were included if they compared NAC versus Surgery Alone (SA) in patients with GC and at least one of following reported outcomes: resectability, OS, PFS, year-specific survival rate (SR) and recurrence, and Grade 3 or 4 adverse events. Furthermore, all included studies followed a proper RCT design. There was no restriction for language or publication status. Data expressed as medians were not included and case series, case reports, reviews and duplicates were excluded. Finally, studies that reported data comparing outcomes of patients with or without postoperative chemotherapy were excluded.

### Data collection and quality assessment

Two reviewers independently extracted data from eligible studies using a standardized data extraction table. Any disagreement was settled by discussion or, in the absence of a consensus, by a third reviewer. The data collected included the first author’s name, country, publication year, number of participants, mean age, percentage male, disease status, NAC chemotherapy regimen, and design of trials included. Reported outcomes included resectability, OS, PFS, 1-year SR, 2-year SR, 3-year SR, 4-year SR, 5-year SR, 1-year recurrence rate, 2-year recurrence rate, 3-year recurrence rate, 4-year recurrence rate, 5-year recurrence rate, and Grade 3 or 4 adverse events. The quality of the eligible studies was evaluated using the Jadad scale [15]. Randomization, blinding, withdrawals, generation of random numbers, and concealment of allocation as the essential parts to a RCT, were scored ranged 0 to 5. A threshold of ≥4 points was regarded as a high-quality study. Any inconsistencies were solved by group discussion for a consensus.

### Statistical analysis

We assigned the results of each RCT as dichotomous frequency data. Relative risks (RR) and 95% confidence intervals (CI) were calculated for each study from event numbers and total patients extracted from each trial before data pooling. The overall HR or RR and 95% CI of resectability, OS, PFS, 1-year SR, 2-year SR, 3-year SR, 4-year SR, 5-year SR, 1-year recurrence rate, 2-year recurrence rate, 3-year recurrence rate, 4-year recurrence rate, 5-year recurrence rate, and Grade 3 or 4 adverse events were also calculated. Both fixed-effect and random-effect models were used to evaluate the pooled HR or RR for patients who received NAC compared with patients with surgery alone. Although both models yielded similar findings, results from the random-effect model, which assumes that the true underlying effect varies among included trials, are presented here [16, 17]. Sensitivity analysis was conducted by removing each individual study from the meta-analysis [18]. Subgroup analyses were conducted for resectability, OS and PFS on the basis of country, mean age, percentage male, percentage of tumor stages (I and II), and disease status. The Egger [19] and Begg tests [20] were also used to statistically assess publication bias for each outcome. All reported *P* values are 2-sided, and P values < 0.05 were considered statistically significant for all included studies. Statistical analyses were performed using STATA software (version 12.0; Stata Corporation, College Station, TX, USA).

## Results

**Fig. 1:**
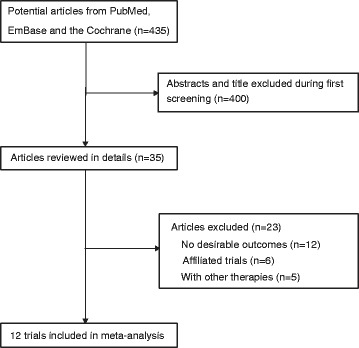
Flow diagram of the literature search and trial selection process

The results of our study selection process are shown in Fig. 1. We identified 435 articles in our initial electronic search, of which 400 were excluded as duplicates or irrelevant studies. A total of 35 potentially eligible studies were selected for further judging. After detailed evaluations, 12 RCTs were selected for the final meta-analysis of the efficacy and safety of NAC and SA [7, 9, 10, 21–29]. A manual search of the reference lists of these studies did not yield any new eligible studies. The general characteristics of the included studies are presented in Table 1.

**Table 1:** Baseline characteristics of studies included in the final meta-analysis

First author	Publication year	Country	Age	Male (%)	Sample size			Chemotherapy regimen	Disease status	Tumor stage (I and II)	Jadad score
					NAC	SA	Total				
Cunningham [21]	2006	UK	62.0	78.7	250	253	503	Cisplatin; fluorouracil	Resectable Gastroesophageal Cancer	43.8%	5
Hartgrink [22]	2004	Netherland	NG	NG	27	29	56	Methotrexate; 5-fluorouracil; leucovorin	Resectable GC	53.6%	4
Hashemzadeh [23]	2014	Iran	59.2	75.7	22	52	74	Docetaxel; cisplatin; 5-fluorouracil	Locally advanced GC	28.0%	1
Lygidakis [24]	1999	Greece	61.0	47.4	19	19	38	Mitomycin C; 5-fluorouracil; leucovorin; farmorubicin	Resectable GC	36.8%	2
Qu [25]	2010	China	56.0	61.5	39	39	78	Docetaxel	Advanced GC	0.0%	3
Schuhmacher [10]	2010	Europe	57.0	69.4	72	72	144	Cisplatin; fluorouracil	Locally Advanced Cancer of the Stomach and Cardia	0.0%	3
Sun [26]	2011	China	NG	NG	29	26	55	Docetaxel; dexamethasone; cimetidine; phenergan	Borrmann Type IV GC	NG	1
Wang [27]	2000	China	54.5	83.3	30	30	60	5-fluorouracil	Gastric cardia cancer	18.3%	1
Ychou [9]	2011	France	63.0	84.0	113	111	224	Fluorouracil; cisplatin	Resectable Gastroesophageal Adenocarcinoma	31.1%	3
Yonemura [7]	1993	Japan	60.5	74.5	26	29	55	Cisplatin; mitomycin C; etoposide;l-(2-tetrahydrofuryl)-5-fluorouracil; uracil	High-Grade Advanced GC	16.4%	2
Zhang [28]	2012	China	NG	60.0	38	42	80	Calcium folinate; oxaliplatin; 5-fluorouracil	Advanced GC	0.0%	2
Kobayashi [29]	2000	Japan	NG	NG	91	80	171	5-fluorouracil	Resectable GC	NG	2

*NAC* neoadjuvant chemotherapy, *SA* surgery alone, *GC* gastric cancer *NG* not given

The 12 included trials involve a total of 1538 GC patients. The sample sizes ranged from 38 to 503, with mean ages ranging from 54 to 64 years. Five trials were conducted in Europe [9, 10, 21, 22, 24], and the remaining 7 were conducted in Asia [7, 23, 25–29]. Study quality was evaluated using the Jadad scale. Overall, 1 trial [21] had a score of 5, 1 trial [22] had a score of 4, 3 trials [9, 10, 25] had a score of 3, 4 trials [9, 24, 28, 29] had a score of 2, and the remaining 3 trials [23, 26, 27] had a score of 1.

**Fig. 2:**
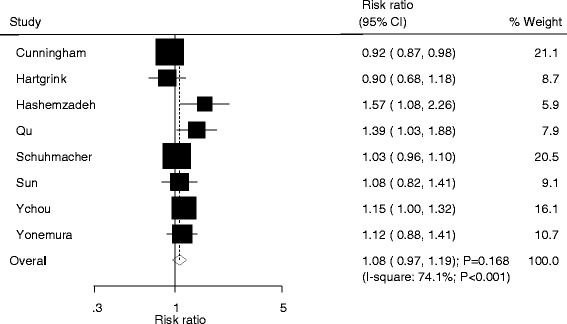
Forest plot showing the relative risk in the resectability between NAC and SA

Data for the effect of NAC on the incidence of resectability were available from 8 trials. The summary RR showed no significant difference between NAC and SA for resectability (RR: 1.08; 95%CI: 0.97–1.19; *P* = 0.168; Fig. 2). Substantial heterogeneity was detected across included trials (*P* < 0.001). As a result, a sensitivity analysis was conducted for resectability and, after excluding Cunningham et al.’s trial which specifically included patients with gastroesophageal cancer, we noted that patients receiving NAC were associated with a non-significant increase in the incidence of resectability (RR: 1.12; 95%CI: 1.00–1.26; *P* = 0.058).

**Fig. 3:**
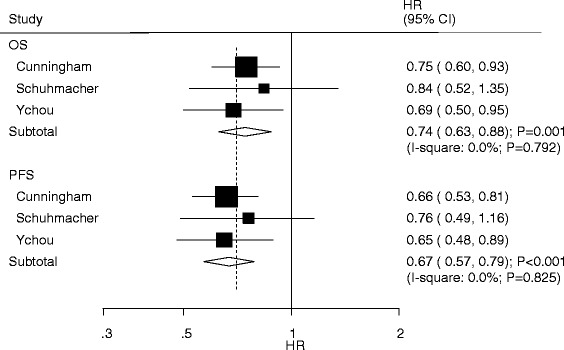
Forest plot showing the hazard ratio in OS and PFS between NAC and SA

Data for OS and PFS were available from 3 trials. NAC was associated with a statistically significant improvement in OS (HR: 0.74; 95%CI: 0.63–0.88; *P* = 0.001; Fig. 3) and PFS (HR: 0.67; 95%CI: 0.57–0.79; *P* < 0.001) as compared with SA. There was no significant heterogeneity across the included trials. Sensitivity analyses were conducted with the sequential exclusion of each trial, with no effect on the conclusions for OS and PFS.

**Fig. 4:**
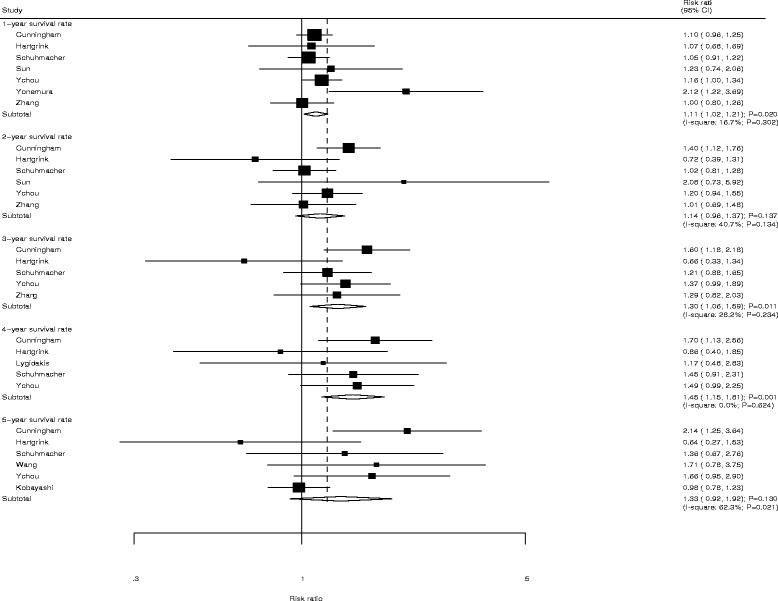
Forest plot showing the relative risk in 1-year SR, 2-year SR, 3-year SR, 4-year SR, and 5-year SR between NAC and SA

Data for the effects of NAC on the incidence of year-specific SR were organized by increased SR per year and listed in Fig. 4. The combined RR suggests that patients who received NAC experienced a significantly increased incidence of 1-year SR (RR: 1.11; 95%CI: 1.02–1.21; *P* = 0.020), 3-year SR (RR: 1.30; 95%CI: 1.06–1.59; *P* = 0.011), and 4-year SR (RR: 1.45; 95%CI: 1.15–1.81; *P* = 0.001). However, there was no significant effect on the incidence of 2-year SR (RR: 1.14; 95%CI: 0.96–1.37; *P* = 0.137), and 5-year SR (RR: 1.33; 95%CI: 0.92–1.92; *P* = 0.130). Moderate heterogeneity was detected in 2-year SR and 5-year SR, while negligible heterogeneity was observed in 1-year SR, 3-year SR, and 4-year SR.

**Fig. 5:**
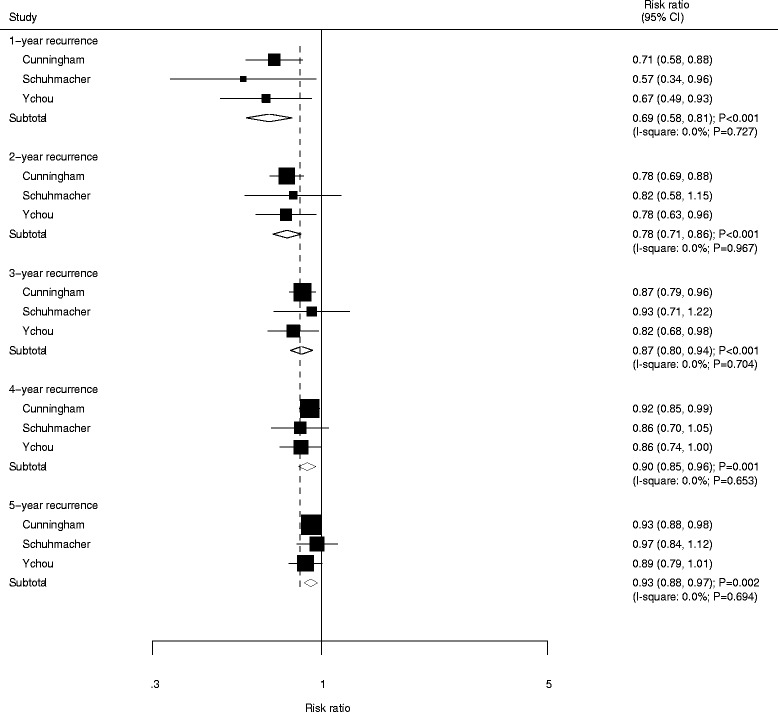
Forest plot showing the relative risk in 1-year recurrence, 2-year recurrence, 3-year recurrence, 4-year recurrence, and 5-year recurrence between NAC and SA

Data for the effects of NAC on the incidence of year-specific recurrence rate were grouped by increased recurrence rate per year and listed in Fig. 5. We noted that patients who received NAC had a significantly reduced risk of 1-year (RR: 0.69; 95%CI: 0.58–0.81; *P* < 0.001), 2-year (RR: 0.78; 95%CI: 0.71–0.86; *P* < 0.001), 3-year (RR: 0.87; 95%CI: 0.80–0.94; *P* < 0.001), 4-year (RR: 0.90; 95%CI: 0.85–0.96; *P* = 0.001), and 5-year recurrence rate (RR: 0.93; 95%CI: 0.88–0.97; *P* = 0.002). There was no significant heterogeneity detected across the included trials.

The combined results of WHO grade 3 or greater adverse events are presented in Table 2. Most specific adverse events were non-significant due to the low number of trials reporting this information. We noted that patients who received NAC were associated with an elevated risk of developing lymphocytopenia (RR: 2.02; 95%CI: 1.27–3.24; *P* = 0.003), and hemoglobinopathy (RR: 11.13; 95%CI: 1.45–85.58; *P* = 0.021) when compared with SA. No significant effect was detected across the included trials for other adverse events.

**Table 2:** Summarized of grade 3 or greater adverse events

Outcomes	NAC group	Control group	RR (95% CI)	P value	P value for heterogeneity
Granulocytopenia	67/222	52/240	1.23 (0.81–1.87)	0.338	0.209
Lymphocytopenia	46/204	23/230	2.02 (1.27–3.24)	0.003	–
Leukopenia	39/250	29/263	1.26 (0.59–2.66)	0.552	0.086
Hemoglobinopathy	11/239	1/252	11.13 (1.45–85.58)	0.021	–
Thrombocytopenia	2/287	6/286	0.35 (0.07–1.72)	0.195	0.677
Other hematologic abnormality	1/249	2/251	0.51 (0.05–5.55)	0.577	–
Nausea	25/264	29/263	0.86 (0.53–1.41)	0.559	0.889
Vomiting	13/237	14/239	0.94 (0.45–1.96)	0.868	–
Neurologic effects	10/279	7/285	1.46 (0.55–3.89)	0.453	0.329
Skin effects	8/242	2/251	4.05 (0.87–18.88)	0.075	–
Stomatitis	10/240	5/248	2.02 (0.70–5.84)	0.192	–
Diarrhea	6/244	5/248	1.21 (0.38–3.93)	0.746	–

Subgroup analyses were performed for resectability, OS, and PFS to evaluate the effect of NAC in specific subpopulations (Table 3). First, we noted NAC was associated with higher resectability if the patients included in individual trial were Asian. Second, patients who received NAC has no significant effect on OS if the mean age of patients less than 60, percentage male less than 70%, percentage of tumor stage (I and II) less than 30%, and patients with GC. Third, NAC was not associated with PFS if the mean age of patients less than 60, percentage male less than 70%, percentage of tumor stage (I and II) less than 30%, and patients with GC.

**Table 3:** Subgroup analysis

Outcomes	Group	RR (95% CI)	P value	P value for heterogeneity
Resectability	Country			
	Asian	1.23 (1.04–1.46)	0.015	0.243
	Europe	1.00 (0.91–1.11)	0.950	0.008
	Mean age (years)			
	60 or more	1.02 (0.81–1.29)	0.842	0.002
	< 60	1.29 (0.84–1.97)	0.251	< 0.001
	Percentage male (%)			
	70 or greater	1.13 (0.87–1.46)	0.355	< 0.001
	< 70	1.14 (0.91–1.42)	0.257	0.020
	Percentage of tumor stage (I and II) (%)			
	30 or greater	0.99 (0.83–1.18)	0.934	0.009
	< 30	1.22 (0.94–1.59)	0.132	0.001
	Disease status			
	Gastroesophageal cancer	1.02 (0.81–1.29)	0.842	0.002
	Gastric cancer	1.12 (0.96–1.31)	0.136	0.018
OS	Country			
	Asian	–	–	–
	Europe	0.74 (0.63–0.88)	0.001	0.792
	Mean age (years)			
	60 or more	0.73 (0.61–0.88)	0.001	0.674
	< 60	0.84 (0.52–1.35)	0.474	–
	Percentage male (%)			
	70 or greater	0.73 (0.61–0.88)	0.001	0.674
	< 70	0.84 (0.52–1.35)	0.474	–
	Percentage of tumor stage (I and II) (%)			
	30 or greater	0.73 (0.61–0.88)	0.001	0.674
	< 30	0.84 (0.52–1.35)	0.474	–
	Disease status			
	Gastroesophageal cancer	0.73 (0.61–0.88)	0.001	0.674
	Gastric cancer	0.84 (0.52–1.35)	0.474	–
PFS	Country			
	Asian	–	–	–
	Europe	0.67 (0.57–0.79)	< 0.001	0.825
	Mean age (years)			
	60 or more	0.66 (0.55–0.78)	< 0.001	0.936
	< 60	0.76 (0.49–1.17)	0.212	–
	Percentage male (%)			
	70 or greater	0.66 (0.55–0.78)	< 0.001	0.936
	< 70	0.76 (0.49–1.17)	0.212	–
	Percentage of tumor stage (I and II) (%)			
	30 or greater	0.66 (0.55–0.78)	< 0.001	0.936
	< 30	0.76 (0.49–1.17)	0.212	–
	Disease status			
	Gastroesophageal cancer	0.66 (0.55–0.78)	< 0.001	0.936
	Gastric cancer	0.76 (0.49–1.17)	0.212	–

The Egger and Begg test results showed no evidence of publication bias for resectability, OS, PFS, 1-year SR, 2-year SR, 3-year SR, 4-year SR, 1-year recurrence, 2-year recurrence, 3-year recurrence, 4-year recurrence, or 5-year recurrence. Although the Begg test showed no evidence of publication bias for 5-year SR (*P* = 0.452), the Egger test showed potential evidence of publication bias for 5-year SR (*P* = 0.009) (Table 4). The conclusion was unchanged after adjustment for publication bias by using the trim and fill method [30].

**Table 4:** Publication bias

Outcomes	P value for Egger	P value for Begg
Resectability	0.363	0.711
OS	0.763	1.000
PFS	0.444	1.000
1-year survival rate	0.376	0.764
2-year survival rate	0.266	1.000
3-year survival rate	0.991	0.806
4-year survival rate	0.065	0.221
5-year survival rate	0.009	0.452
1-year recurrence	0.069	1.000
2-year recurrence	0.242	0.296
3-year recurrence	0.243	0.296
4-year recurrence	0.723	1.000
5-year recurrence	0.887	1.000

## Discussion

This meta-analysis of studies analyzing the efficacy and safety of NAC included updated data from previously published studies and additional new RCTs not reviewed in previously published works. This additional information allows for a more robust analysis of the effect of NAC on survival outcomes for GC. The results of this updated meta-analysis indicate that NAC could elicit improvements in OS, PFS, 1-, 3-, and 4-year SR, and 1-, 2-, 3-, 4-, and 5-year recurrence in treatment of patients with GC as compared with those received SA. Conversely, patients receiving NAC also experienced a significantly increased risk of developing lymphocytopenia, and hemoglobinopathy. No other significant differences were detected across included trials.

The methodological assessment of individual trial was the essential parts including randomization, blinding, withdrawals, generation of random numbers, and concealment of allocation. This meta-analysis provides clear information about randomization and withdrawals, whereas other forms were available in few trials and might contribute to heterogeneity in overall analysis. Therefore, we critically this recommendations for the treatment of patients with GC due to the unsatisfactory quality of included trials.

There were certain limitations present in previous meta-analysis articles exploring the efficacy and safety of NAC on survival outcomes for gastric carcinoma.. Liao et al. [6] suggested that NAC was associated with an insignificant increase in the incidence of overall survival, R0 resection, postoperative complications, and perioperative mortality. Furthermore, Xiong et al. [31] conducted an updated meta-analysis of RCTs and found that NAC can significantly improve SR, 3-year PFS, tumor down-staging rate and R0 resection rate, whereas it had no significant effect on relapse rates, operative complications, perioperative mortality and grade 3/4 adverse events. However, these studies did not report year-specifically SR and recurrence. Additionally, although several trials suggest that NAC can be used as a standard therapy for patients with GC, the superiority of NAC over SA remains unclear due to the greater adverse events detected in the NAC group. Therefore, it was necessary to conduct an updated meta-analysis to explore further information regarding the efficacy and safety of the NAC in treatment of patients with GC.

There was no significant overall difference for the incidence of resectability between NAC and SA groups. However, three trials included in our study reported inconsistent results. The MAGIC Trial [21] suggested that patients with resectable gastroesophageal cancer who received NAC were associated with a lower incidence of resectability, whereas two other trials [23, 25] indicated that NAC therapy significantly increased the incidence of resectability. A possible explanation could be that patients who received NAC therapy might have had their surgery postponed, allowing for the disease to progress, causing these patients to lose a chance to undergo curative surgery.

The findings of our study suggest that patients who received NAC therapy experienced significant improvement in OS, PFS, and year-specifically SR and recurrence, although there was no significant difference between NAC and SA for 2-year SR and 5-year SR. The cause of this could simply be the smaller number of trials reporting these outcomes. Further, the reason for no significant difference for 5-year SR might affected by the Kobayashi et al.’s study, which included patients received the low dose of 5′-deoxy-5-fluorouridine. Furthermore, the use of NAC was considered in order to lower the stage of the tumor and improve resectability and survival. Therefore, NAC might play a beneficial role in the treatment of patients with GC.

As expected, NAC therapy was associated with an increased risk of some toxicity. The improvement of survival outcomes should balance these risks if used on grade 3 or greater adverse events, which optimize the impact on the patients’ quality of life. However, data on specific adverse events were rarely available and these results may be variable due to the low number of trials included. Therefore, we only aim to provide a synthetic and comprehensive review for adverse events in aggregate.

In our study, patients received NAC was associated with a higher incidence of resectability when the study included Asians. These findings were inconsistent with the study included Europeans. This could be because the percentage of tumor stage (I and II) was higher in Europe, which associated with higher resectability rate. Further, the tumor stages was higher in Asia than Europe, and the treatment effect on resectability was obvious. Two of included trials provided higher weight (21.1% and 20.5%; Fig. 2) were conducted in Europe and reported no significant effect on resectability, which could affect the treatment effect of NAC on resectability to no statistically significant [21]. In addition, disease status, tumor stages were also play an important role on treatment effect. Although no significant difference were detected, the reason could be that the analysis included smaller patient cohorts, and the result may be unstable. Furthermore, the results of subgroup analyses for OS and PFS were restricted due to only three trials provided the data of OS and PFS.

Two strengths of our study should be highlighted. First, the large sample size allowed us to quantitatively assess the efficacy and safety of NAC in the treatment of GC patients, thus our findings are potentially more robust than those of any individual study. Second, we specifically reported year-specific SR and recurrence, and summarized grade 3 or greater adverse events, which allows for an accurate assessment of the benefits and harms for GC patients.

The limitations of our study are as follows: (1) in a meta-analysis of published studies, publication bias is an inevitable problem; (2) the analysis used pooled data (individual data were not available), which restricted us from performing a more detailed relevant analysis and obtaining more comprehensive results; (3) data on adverse events or quality of life were rarely available in included trials, so the conclusion may be variable; and (4) In the planning stages, we intend conducted subgroup analyses based on gender (men, women), and tumor stages (I, or II, and III or IV), whereas the results of stratified analysis in individual trial were not available.

## Conclusions

The findings of this study indicate that NAC might play an important role on the outcomes of survival rate and disease progression for patients with GC. However, it may also associate with an increased risk in for adverse effects. Future trials should focus on specific disease status and record pre- and post-operative adverse events.

## Abbreviations

GC: Gastric cancer
NAC: Neoadjuvant chemotherapy
RCTs: Randomized controlled trials
SR: Survival rate
